# S1P in Tumor Microenvironment and Modulation of Anti-Tumor-Directed T-Cell Responses

**DOI:** 10.3390/cells15100909

**Published:** 2026-05-15

**Authors:** Patrícia A. António, Joana R. Lérias, Carolina M. Gorgulho, Karina Balan, Vitaly Balan, Markus J. Maeurer

**Affiliations:** 1 Immunotherapy/ImmunoSurgery Laboratory, Cell Center at the Champalimaud Foundation, Avenida Brasília, 1400-038 Lisbon, Portugal; patricia.antonio@research.fchampalimaud.org (P.A.A.); joana.lerias@research.fchampalimaud.org (J.R.L.); carolina.gorgulho@research.fchampalimaud.org (C.M.G.); karina.balan@research.fchampalimaud.org (K.B.); vitaly.balan@research.fchampalimaud.org (V.B.); 2 I Med Clinic, Johannes Gutenberg University of Mainz, 55131 Mainz, Germany

**Keywords:** tumor-infiltrating lymphocytes, S1P, sphingosine-1-phosphate receptor, T-cell receptor, CD69

## Abstract

Adoptive cell therapy (ACT) using tumor-infiltrating lymphocytes (TILs) has achieved clinically and biologically relevant responses in patients with solid cancer. Clinical efficacy has been increasingly linked to a specific T-cell phenotype, particularly CD8^+^ TILs exhibiting a progenitor stem-cell-like profile (CD39^−^ CD69^−^). This review explores the critical role of the sphingosine-1-phosphate (S1P) axis in orchestrating these responses. We detail the biological antagonism between the activation marker CD69 and S1P receptor 1 (S1PR1), where mutual exclusivity dictates thymic selection, if T-cells are retained in tissues or allowed to recirculate and maintain long-term immune surveillance. The S1PR1:S1P axis is further recognized as a critical regulator of mitochondrial fitness, sustaining the high energetic demands of precursor T-cells. We examine the “double-edged sword” nature of S1P in the tumor microenvironment (TME), where it can drive pro-tumorigenic processes like angiogenesis and vascular mimicry (VM), be hijacked by cancer cells to create immune-excluded environments, or S1P can increase T-cell fitness. We summarize the current landscape of clinical trials (as of January 2026) that target S1P production or signaling to modulate anti-tumor responses or use S1P as a biologically relevant marker of treatment outcome.

## 1. Introduction

Adoptive cell therapy (ACT) has achieved clinically and biologically relevant responses in patients with solid cancer, and 2024 witnessed the first FDA approval of tumor-infiltrating lymphocytes (TILs) for patients with metastatic melanoma [1]. Phenotypic flow cytometric analyses are used to assess the release of TIL products prior to therapy. These include IFN-γ production in response to T-cell receptor (TCR) cross-linking [2,3,4,5,6] as well as exploratory assays to map the immune reactivity of TIL [7,8] to private mutations, which have been described as targets for clinically relevant immune responses in patients with solid cancer. This was first described by Wolfel and coworkers for T-cells from a patient with melanoma targeting a CDK4 point mutation [9]. Other tumor antigens associated with clinically relevant responses have also been described—non-mutant tumor-associated antigens, such as Melan-A/MART-1, gp100, or tyrosinase—for patients with melanoma [10]; cancer–testis antigens, such as NY-ESO-1 [11]; or antigens expressed at low levels in healthy tissue, yet overexpressed in cancer, such as mesothelin [12] or PRAME [13]. The latter antigen is now being successfully explored for transgenic TCR-IL-15-NK cells expressing a TCR that targets PRAME in patients with HLA-A*02:01-positive relapsed and/or refractory metastatic melanoma [14]. Target antigens for gauging TIL reactivity can be provided by synthetic peptides [15,16,17,18,19], or cancer mutations can be delivered by viral vector systems into antigen-presenting cells [20] and subsequently tested for T-cell recognition. The “dark matter” in cancer cells, recognized by cancer-specific T-cells, may be more challenging to define, since such tumor targets may be short-lived [21,22,23]. Here, whole DNA sequencing [24,25] as well as mass spectrometry of peptides eluted from major histocompatibility complex (MHC) molecules may be required [23]. ACT utilizes ex vivo expanded TILs that are most likely clinically effective if they contain T-cells targeting private mutations, such as point mutations, frameshifts, indels, or misfolded proteins. Mutations do not necessarily imply immunogenicity: (i) proper processing and presentation of potential tumor-associated targets by the antigen processing and presentation machinery is required [26]; (ii) processing of tumor targets picked up by professional antigen-presenting cells and tumor cells may be different [27]; and (iii) antigen presentation depends on the MHC genetic background, i.e., the *KRAS G12V* mutation is presented by HLA-A*3, A*11, and C*08:02 [28] and the available TCR repertoire capable of responding to a nominal cancer target [29]. A number of assays have been developed to assess T-cell activation, e.g., by measuring the upregulation of T-cell activation markers (e.g., CD137) or cytokine production [30,31,32,33]. A different, not mutually exclusive approach, attempted to link T-cell phenotypes with recent tumor–target interaction and cancer-specificity [34]. This was shown for PD-1^+^ TIL in patients with melanoma, which could also be identified in the peripheral blood of patients with cancer and enriched for anti-cancer-directed T-cell clones [35]. Most of the data have been obtained from patients with melanoma, yet identical mechanisms have been shown to be true for TILs from patients with other tumor types, e.g., TILs from patients with head and neck cancer [36]. Not only CD69 expression, but also a combination of cell surface markers may indicate that T-cells have encountered their nominal target antigen(s). TIL, which expresses CD8^+^ PD-1^+^ and tissue-homing marker CD103, has been proposed to reflect tumor-reactive T-cells in patients with lung cancer. A landmark publication from Krishna et al. showed in 2020 that two distinct T-cell phenotypes are associated with clinical outcome in patients with melanoma: (i) CD8^+^ TIL with progenitor “stem-cell-like” phenotype that mediated complete regression in patients with melanoma and ii) differentiated TIL, enriched for neoantigen-specific T-cells that showed poor persistence in patients, most likely due to their terminally differentiated state. The former TIL population, conferring clinical responses, is of great clinical interest and was found to exhibit a CD8^+^ CD39^−^ CD69^−^ phenotype. This precursor phenotype may present a viable source for identifying and harvesting TILs enriched for anti-cancer reactivity—TILs containing anti-cancer-directed T-cell clones that have not yet been expanded in vivo but still retain the possibility for a long-term protective cellular immune response upon and in subsequent antigen encounters [37].

Of particular interest is the phenotype of anti-cancer-directed TILs, particularly T-cells with a CD69^−^ precursor (CD45RA^+^CCR7^+^) phenotype. CD69 is one of three markers upregulated upon antigen exposure, along with CD25 and HLA-DR [31]. While a T-cell is CD69^−^, it may express the sphingosine-1-phosphate (S1P) receptor 1 (S1PR1). The expression of CD69 and S1PR1 is mutually exclusive, i.e., CD69^−^ TILs are S1PR1^+^. Not only in peripheral or tissue-resident T-cells that display a broad range of differentiation and maturation, but also in thymic editing, CD69 and S1PR1 play a vital role in determining T-cell fate. Immune progenitor cells, after entering the thymus, differentiate into CD4^−^ or CD8^−^ -expressing T-cells. For that differentiation to occur, immune cells need to be retained within the thymus via a mechanism involving S1PR1 and CD69. CD69 on the cell surface binds to helix 4 of the S1PR1, preventing extracellular expression. This binding stabilizes the S1PR1 conformation and mimics the binding of the natural ligand, S1P, leading to subsequent internalization and degradation. The reverse situation has also been found to be true: S1PR1 expression prevents surface CD69 expression, even when CD69 is available intracellularly, suggesting an antagonistic interaction between CD69 and S1PR1. CD69 and S1PR1 have been found to co-immunoprecipitate, most likely due to post-translational modifications, suggesting an evolutionarily conserved, physiologically relevant pathway between S1P:S1PR and CD69 in T-cell differentiation and activation [38,39,40,41,42]. In summary, a “young” CD8^+^ CD69^−^ T-cell phenotype, associated with T-cell precursors, is thought to be clinically advantageous, and the CD8^+^ CD69^−^ T-cell population exhibits most likely an S1PR1^+^ phenotype. The association between CD69^−^ and S1PR1^+^ in TILs has not, to our knowledge, been reviewed until today. We therefore address, in this review, the biology and physiological role of sphingosine-1-phosphate (S1P) and its receptor(s), the role of S1P in the tumor microenvironment (TME), and ongoing clinical trials targeting S1P and S1P receptors.

## 2. Sphingosine-1-Phosphate (S1P) Characterization

**Figure 1:**
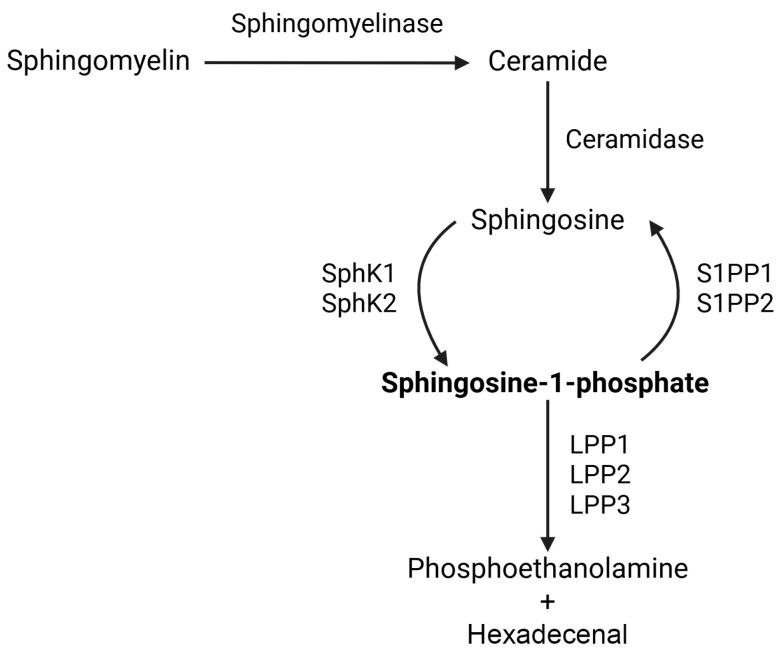
Pathway of S1P production, degradation, and metabolic intermediate metabolites. Sphingomyelin is converted into ceramide by sphingomyelinase, which is subsequently converted into sphingosine by ceramidase. Sphingosine can further be phosphorylated by sphingosine kinase 1 (SphK1) or sphingosine kinase 2 (SphK2), leading to the formation of sphingosine-1-phosphate (S1P). This phosphorylation status can be reversed by sphingosine-1-phosphate phosphatase 1 (S1PP1) or sphingosine-1-phosphate phosphatase 2 (S1PP2), creating a loop between sphingosine and S1P. S1P can also be converted into phosphoethanolamine and hexadecenol by the action of lipid phosphate phosphatase 1 (LPP1), lipid phosphate phosphatase 2 (LPP2), or lipid phosphate phosphatase 3 (LPP3).

Sphingolipids are crucial for cell membrane structure and function, with a core composition of a sphingoid base, sphingosine, a fatty amino alcohol with 18 carbon atoms, which enables the formation of polar lipid mediators [43,44]. S1P is a derivative of sphingosine that orchestrates T-cell physiology, mitochondrial fitness, and T-cell homing [45] (Figure 1).

S1P signals either in a receptor-dependent manner or by receptor-independent recognition [46]. S1P can be characterized by its “inside-out signaling” capacity, due to its autocrine and paracrine actions: S1P mediates biological responses either intracellularly (receptor-independent) or extracellularly (receptor-dependent). S1P may also signal to adjacent cells via receptor engagement in a paracrine manner [38,45,47].

Healthy individuals exhibit a characteristic S1P gradient in the peripheral circulation and in tissues, maintained by a balance between S1P production and degradation. This S1P gradient allows “S1P-gradient-dependent” immune responses [42,45]. S1P levels are high in blood and lower in the lymphatic system, followed by the lowest S1P concentration in tissues. Levels of S1P-degrading enzymes are responsible for maintaining that gradient; i.e., they are elevated in tissue, thereby limiting S1P accumulation. S1P can be produced by almost every cell type: in blood, S1P is provided by erythrocytes, while endothelial cells provide S1P in the lymphatic system and in tissues [38,40]. Inflammation may change S1P production, thereby facilitating the recruitment of immune cells into tissues [45].

S1P-mediated signaling affects many aspects of cellular homeostasis, including cell proliferation, survival, migration, differentiation, and immune cell activation [45,48,49]. S1P signaling orchestrates lymphocyte differentiation during embryonic development, and it affects endothelial barrier integrity, cytokine adhesion molecule expression, angiogenesis, cardio-genesis, limb development, and neurogenesis [38,39,43]. S1P binds to its nominal cell surface receptors in the extracellular milieu, leading to the activation of anti-apoptotic BCL-2 and MCL1 pathways, and it decreases the expression of pro-apoptotic proteins, i.e., BAD and BAX [50]. S1P also exhibits a broad array of intracellular functions, i.e., S1P acts as a second messenger in multiple signaling pathways, including TRAF2, ceramide synthases, Akt, and STAT5 [39,42,51].

### 2.1. S1P Transport and Receptors

The characteristic polar S1P head does not allow cross-cell transport across membranes, requiring specific transporters, e.g., Spinster 2 (SPNS2) or members of the ABC transporter family (ABCA1, ABCC1, and ABCG2) [52,53,54]. Intracellular S1P can engage directly with its intracellular targets, either by modulating signaling pathways or via engagement with (intracellular) S1P receptors. This duality of S1P, allowing it to act both intracellularly and extracellularly, is called “inside-out signaling,” a distinct characteristic of S1P that can act in a paracrine or autocrine fashion [52].

S1P binds to a series of five G-protein-coupled receptors (GPCRs), i.e., the S1P receptors 1,2,3,4,5 (S1PR1-5) [43,50,53]. These five receptors are members of the rhodopsin subfamily of GPCRs, featuring an intracellular C-terminus, seven helical transmembrane domains, and an extracellular N-terminus with 30 to 50 residues [55]. S1P engagement to its receptors leads to the activation of G-proteins such as Gαq, Gαi, and Gα12/13 [48,53].

S1PR was first characterized by Hla and Macaig in 1990 and was labeled as an endothelial differentiation gene-1 (EDG-1) [56]. It was later renamed S1PR1, followed by the characterization of four more receptors (S1PR2–5) [55].

Most cells express more than one S1PR subtype on their surface. For instance, 83 to 98% of T-cells in peripheral blood express S1PR1, 1 to 15% express S1PR3, 30 to 50% express S1PR4, and 5 to 25% express S1PR5 [43]. Thus, S1P activity is mediated by the repertoire of S1P receptors on responding cells [50].

### 2.2. Physiological Effects of S1P Receptor Engagement

#### 2.2.1. S1PR1

S1PR1 plays a crucial role in immune cell function, including cell trafficking, proliferation, and maturation/differentiation [52]. S1PR1 is coupled to the alpha subunit of Gi proteins, which initiates pathways such as Ras/ERK, NLRP3/IL-1B, PI3K/Akt-mTOR, PI3K/Rac, STAT3, PLC, MAPK, and cAMP [48,55].

Recent structural and functional work supports a more specific mechanism in which CD69 acts in cis as a protein agonist of S1PR1. The CD69 transmembrane helix contacts S1PR1 transmembrane helix 4 and allosterically shifts S1PR1 transmembrane helices 5-6 to engage the heterotrimeric Gαi complex, followed by Gαi-dependent S1PR1 internalization, leading to a loss of S1P gradient sensing and inhibition of lymphocyte egress [57].

A biologically relevant activity of S1P:S1PR1 signaling is the maintenance of mitochondrial function, T-cell fitness, and survival. Precursor T-cells depend on oxidative phosphorylation to meet their high energy demands. A loss of S1PR1 signaling leads to PINK1 accumulation and ubiquitination of mitochondrial proteins, resulting in mitochondrial dysfunction and decreased numbers of immune cells [58].

The S1P:S1PR1 axis facilitates the trafficking and migration of various immune populations, including T-cells, B-cells, dendritic cells, macrophages, neutrophils, hematopoietic progenitors, mast cells, and osteoclasts [38,59]. S1P:S1PR1 signaling guides T-cell maturation and thymocyte egress from the lymph nodes after immune activation, as well as retention in non-lymphoid tissues [60].

**Figure 2:**
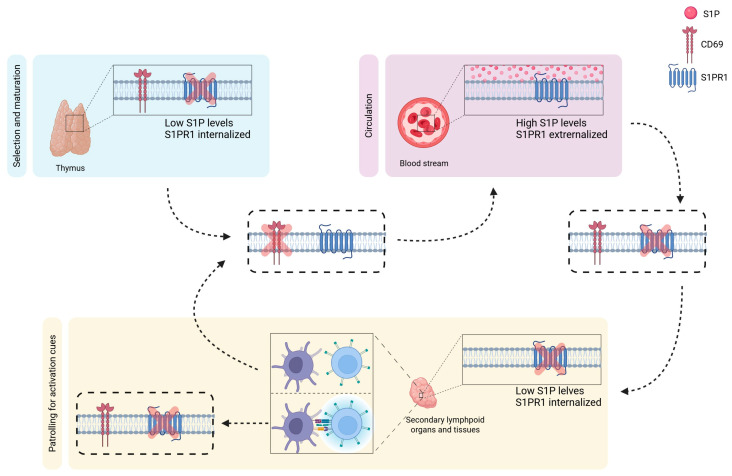
Cell activation in association with S1PR1 expression and immune cell circulation/trafficking. Increased internalization of S1PR1 is associated with retention of immune cells in the thymus and subsequent reduced cell surface S1PR expression, along with transient high CD69 expression. This allows for the process of selection and maturation of T-cells in the thymus (represented by the blue box). Vice versa, increased cell surface S1PR1 expression (and reduced intracellular expression) facilitates tissue egress of immune cells into the circulation. In peripheral circulation (represented by the red box), as T-cells patrol for immune activation cues, S1PR1 expression in the extracellular space is diminished, allowing T-cells to oppose the S1P gradient, i.e., from peripheral circulation into tissues (represented by the yellow box). If T-cells encounter an activation cue, CD69 is upregulated, and S1PR1 expression remains low. Otherwise, S1PR1 is re-expressed on the cell surface, allowing immune cells to return to the circulation. Note the inverse expression of the S1P receptor and CD69, which serves as a marker for tissue-resident cells and T-cell activation. The movements of the T-cells is represented by the arrows.

Early thymic progenitor cells differentiate into CD4^+^ or CD8^+^ T-cells after entering the thymus. To induce T-cell differentiation, immature T-cells must be retained in the thymus via a mechanism involving S1PR1 and CD69. Thymocytes transiently express CD69, which prevents S1PR1 expression on the cell surface (please see the section about CD69 and S1P:S1PR above). Consequently, immature immune cells are trapped in the thymus, facilitating thymic selection and maturation. After maturation, T-cells downregulate CD69 expression and upregulate KLF2, leading to increased surface S1PR1 expression. Immune cells sense the S1P gradient and move out of the thymus into the bloodstream. Once in the bloodstream, T-cells internalize the S1P receptor, thereby diminishing S1PR1 expression on the cell surface. With decreased extracellular S1PR1, immune cells become insensitive to the S1P gradient and can move into tissues to patrol for activating cues and to detect their nominal MHC:peptide targets. Upon activation, T-cells maintain low S1PR1 expression through transcriptional changes and upregulate the activation marker CD69, leading to tissue entrapment. Conversely, if no activation cue is given, T-cells re-express S1PR1 and are again subjected to the S1P gradient, returning to the circulation and restarting the search for activating antigen cues (Figure 2) [38,42,58,60,61]. A loss of S1PR1 expression has been shown to retain cells in the lymph nodes, impeding their egress. The ability to keep lymphocytes in the lymph nodes or to allow their circulation is essential for an effective adaptive immune response [39,52].

**Figure 3:**
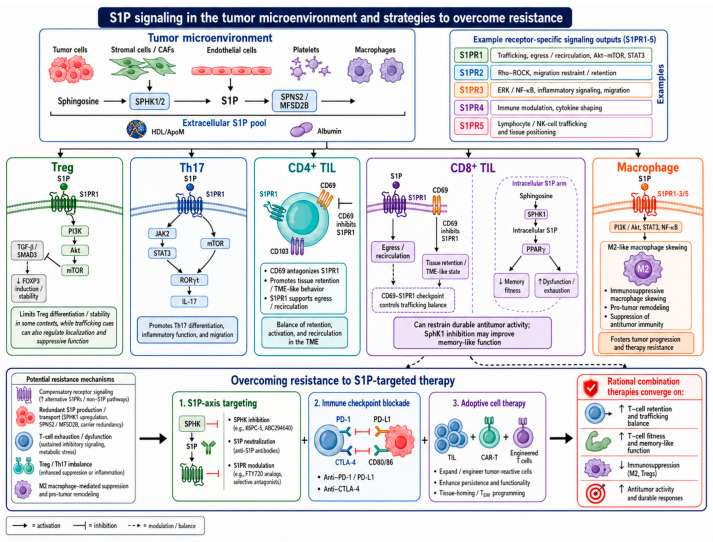
S1P signaling in the tumor microenvironment and therapeutic strategies to overcome treatment resistance. S1P is produced from sphingosine by SphK1/2 and exported through transporters, e.g., SPNS2 and MFSD2B, contributing to the extracellular S1P pool carried by high-density lipoprotein (HDL), apolipoprotein M HDL, and albumin. In the TME, S1P can be supplied by tumor cells, stromal cells, or cancer-associated fibroblasts, endothelial cells, platelets, and macrophages. The figure summarizes representative receptor-specific outputs of S1PR1 to S1PR5 and illustrates how S1P signaling can differentially affect T-reg, Th17, CD4^+^ TILs, CD8^+^ TILs, and macrophage states. In T-cells, S1P-S1PR1 signaling influences trafficking, egress, tissue retention, activation, and differentiation programs. The CD69:S1PR1 axis is a key regulator of TIL retention versus recirculation, while intracellular SphK1-derived S1P may affect CD8^+^ T-cell metabolic programming through PPARγ, contributing to reduced memory fitness and dysfunction. In macrophages, S1P signaling can promote M2-like immunosuppressive polarization and protumor remodeling. The lower panel highlights potential resistance mechanisms to S1P-targeted therapy and proposes rational combinations with immune checkpoint blockade and adoptive cell therapy to improve T-cell fitness, reduce immunosuppression, and enhance durable anti-tumor responses.

To properly execute their physiological and effector functions, distinct immune cell subsets respond differently to S1P. For instance, even though both CD4^+^ and CD8^+^ precursor T-cells in tissues are subject to S1P chemotaxis, the latter show a stronger S1P:S1PR signaling-induced migration (Figure 3). Conversely, human tonsillar effector memory T-cells do not migrate in response to S1P stimulation, indicating a dynamic shift in sensitivity during differentiation. The expression of S1PR1 increases in effector and memory T-cells during T-cell differentiation in order to allow egress from secondary lymphoid organs. Conversely, in tissue-resident memory T-cells, S1PR1 expression is low, and CD69 expression is increased, further inhibiting immune cell migration [60]. S1PR1 signaling also affects regulatory T-cells (T-regs): S1P inhibits T-reg differentiation and suppresses T-reg activity via the Akt-mTOR pathway (Figure 3). S1PR1 signaling can also regulate the switch between T-regs present in non-lymphoid tissues (effector T-regs) and those from lymphoid tissues and the circulation (central T-regs), usually promoting a central T-reg phenotype [42,59].

#### 2.2.2. S1PR2, S1PR3, S1PR4, and S1PR5

S1PR2 is expressed in macrophages, monocytes, granulocytes, and T-cells. While S1PR1 is not expressed during T-cell maturation, S1PR2 expression remains stable from naive to fully matured T-cells. S1PR2 binds to the G12/13 subunit of G-proteins and signals via Rho proteins. The signaling of this receptor can antagonize S1PR1 expression in osteoclast precursors and germinal center B-cells, thereby ensuring cell localization (Figure 3). Intracellular S1P levels activate S1PR2, leading to phosphorylation of ezrin–radixin–moesin (ERM) proteins. These proteins facilitate phagocytic cell functions and phagosome maturation [48,49,60].

S1PR3 can modulate dendritic cell maturation, migration, and endocytosis. The increased activity of professional antigen-presenting cells can therefore modulate adaptive immune cell activation via MHC-restricted antigen presentation. S1P:S1PR3 signaling has also been implicated in macrophage chemotaxis and killing, leukocyte rolling, and immune cell recruitment to inflamed sites as well as bacterial clearance (Figure 3) [48,49].

Knowledge on S1PR4 remains very limited, despite its abundant expression on immune cells. S1P:S1PR4 signaling is most likely related to Rho kinases and cytoskeletal rearrangement, regulation of T-cell proliferation, and cytokine production (Figure 3). Emerging in vivo data support a tumor-promoting role for S1PR4 via restraining CD8^+^ cell expansion. In murine mammary- and colitis-associated colorectal cancer models, genetic ablation of S1PR4 delayed tumor development and improved chemotherapy response by increasing CD8^+^ T-cell abundance, and it modestly improved anti-PD-1 therapy [48,60,62].

S1PR5 is associated with natural killer (NK) cell homing, immune cells’ exit from lymph nodes or the bone marrow and subsequent immune cell recruitment to inflamed organs (Figure 3) [48].

#### 2.2.3. Intracellular S1P, SphK1, and SphK2

S1P can bind to its target prior to exiting the cell, being an important intracellular second messenger [45]. Intracellular S1P antagonizes its precursor, i.e., ceramide, which has been shown to induce apoptosis. While ceramide stimulates the JNK/SAPK pathway and inhibits the ERK and PI3K/Akt pathways, leading to apoptosis, intracellular S1P is a viable inhibitor of apoptosis. Thus, the balance between S1P and ceramide decides cell fate ultimately: apoptosis or prevention of apoptosis [52,63].

Intracellular S1P can bind to the TNF receptor-associated factor 2 (TRAF2), activating the NF-kB pathway. S1P also activates protein kinase C (PKC), leading to NF-κB pathway activation and the subsequent production of pro-inflammatory cytokines such as TNF-α, IL-1, and IL-6 [42,45,63]. TNF-α also stimulates S1P production by activating the kinases ShpK1 and SphK2, creating a loop of anti-apoptotic signaling [50].

In hypoxic conditions, a situation frequently found in tumor lesions, the transcription factors HIF-1a and HIF-2a bind to the SphK1 promoter, resulting in increased intracellular S1P without changes in the S1P extracellular receptor complex. Intracellular S1P can be exported and may act on endothelial cells, fibroblasts, and smooth muscle cells, leading to neovascularization that facilitates tissue oxygenation. Notably, SphK1 may also upregulate HIF-1α, creating a feedback loop that may rescue cells from hypoxia-induced cell death [50,64,65,66].

In the nucleus, S1P and SphK2 can act as epigenetic regulators. S1P binds to and inhibits histone deacetylase 1 (HDAC1) and histone deacetylase 2 (HDAC2). SphK2 orchestrates not only S1P production but also a broader array of genetic and epigenetic functions, e.g., increasing histone H3 acetylation, leading to increased DNA acetylation and repression of gene transcription. SphK2 may also upregulate p21, a cyclin-dependent kinase inhibitor. Since p21 is a cell cycle inhibitor, SphK2 has been associated with cell cycle arrest and apoptosis. In mitochondria, the presence of SphK2 is necessary for the assembly of the cytochrome oxidase complex. In contrast, other studies suggest that SphK2 overexpression leads to cytochrome c release and caspase 3 activation, resulting in apoptosis [50,52,55]. The fine balance of SphK2 levels is therefore essential for immune cell survival and cellular “fitness”.

## 3. Sphingolipids: The Substrate for T-Cell Fitness

### 3.1. Sphingolipids in Mitochondrial Health

Mitochondria can be divided into four compartments: the outer membrane, the inner membrane, the intermembrane space, and the matrix. Each compartment is characterized by specific marker proteins and enzymes. The junctions between the outer and inner membranes may serve as sites for protein import and phospholipid translocation [67].

Mitochondria can autonomously synthesize several lipids, yet they also depend on lipids supplied by the endoplasmic reticulum. A continuous supply and exchange of lipids is necessary to maintain mitochondrial membrane integrity and cellular function. Lipid interactions alter the biophysical properties of mitochondrial membranes, further affecting mitochondrial permeability and their interactions with intracellular factors that regulate and integrate mitochondrial activity into cellular metabolism and cell “fitness” [67,68].

Alterations in sphingolipid levels can modify the morphology of the mitochondrial inner and outer membranes. In turn, alterations in mitochondrial membranes lead to aberrant mitochondrial functions, including cell death, autophagy, chemoresistance, or cellular stress [69].

The balance between two mitochondrial processes, fusion and fission, has implications in cell physiology. Mitochondrial fusion generally generates healthy mitochondria, while fission precedes the removal of non-functional mitochondria through mitophagy. Mitophagy is a selective form of autophagy that enables the elimination of dysfunctional or aged mitochondria, thereby maintaining mitochondrial quality and serving as a mitochondrial quality control mechanism. This balance can be disrupted, leading to consequential effects in cellular “fitness”. Mitochondrial fission can activate a type of programmed cell death, independent of apoptosis, named lethal mitophagy. Lethal mitophagy relies on a dynamin-related protein, called Drp1, that promotes mitochondrial membrane scission [69,70].

Upon stress signals, the outer membrane of mitochondria permeabilizes, allowing proteins from the intermembrane space to be released, thereby activating the caspase cascade and ultimately leading to cell death. One of the critical cellular changes induced by stress is the activation of Drp1, leading to mitochondrial fission and membrane damage. The permeabilization of the outer membrane of mitochondria is an irreversible event and, therefore, considered a critical step in apoptosis [69,71].

S1P binds with high affinity to prohibitin 2 (PHB2), a highly conserved protein in the inner mitochondrial membrane, crucial for the assembly and function of cytochrome c oxidase. A reduced S1P concentration, mediated by SphK2 knockout, has been shown to result in lower oxygen consumption, reduced oxidative phosphorylation via complex IV, and lower membrane potential [72]. This observation further underlined the pivotal role of S1P in mitochondrial integrity and fitness.

In contrast, increased expression of S1P-producing enzymes (including SphK1) was associated with higher mitochondrial calcium levels and increased ATP production. However, excessively high calcium concentrations will also lead to cell death [69].

Sphingolipids can alter mitochondrial membrane curvature, thereby influencing mitochondrial fates, including fusion and fission. Ceramide, a byproduct of sphingosine metabolism, affects mitochondrial morphology by modulating fusion- and fission-associated proteins. S1PR1 overexpression can disrupt the balance between mitochondrial fusion and fission, whereas ceramide induces fission by increasing Drp1 protein expression and oxidative phosphorylation [69,71].

Ceramide can relocate to and accumulate in mitochondria, targeting them for mitophagy. Ceramide production is a physiological cellular response to pro-apoptotic agents. It precedes the mitochondrial phase of apoptosis and is accompanied by the release of mitochondrial intermembrane space proteins, inhibition of the electron transport chain, induction of ROS formation, and a reduction in mitochondrial membrane potential [70,71].

Ceramide can also directly interact with molecules that regulate cellular fitness and survival, such as BAX, BOK, and other BCL-2 family proteins. Ceramide binds to BAX, inducing a conformational change that leads to BAX translocation to the mitochondria and consequent mitochondrial permeabilization via pore formation. Increased ceramide also increases recruitment of BOK to the outer mitochondrial membrane [69,70,71].

In addition to interacting with pro-apoptotic factors, ceramide can accumulate in the outer mitochondrial membrane and trigger permeabilization by forming channels. These channels are essential during apoptosis and allow the passage of mitochondrial components. The formation of ceramide channels can be directly inhibited by the presence of the anti-apoptotic BCL-2 proteins [71,73].

### 3.2. Sphingolipids in the Endoplasmic Reticulum

The endoplasmic reticulum (ER) is a complex tubular organelle that can be divided into the rough ER, which contains ribosomes associated with the membrane and is responsible for protein synthesis, and the smooth ER, which contains very few or no ribosomes and is mainly responsible for lipid production. Protein folding and “quality control” via ubiquitination for proteasomal degradation also occur in the ER. In cancer, several cues from the microenvironment, such as hypoxia, pH alterations, ROS generation, and nutrient deprivation, among others, can disrupt the finely tuned regulation of protein synthesis, folding, and modification in the ER, leading to the accumulation of defective proteins, a condition known as ER stress [74,75,76]. The balance between S1P and ceramides, and perhaps more biologically relevant, the subcellular localization of S1P and its receptors, seems to play an important role in ER stress responses and the induction of cell death. Maceyka and colleagues have shown that serum starvation leads to SphK2 (but not SphK1) localization to the ER in murine fibroblasts and human embryonic kidney cell lines, where SphK2 catalyzes S1P production, which in turn activates a Ca^2+^-mobilization-dependent apoptotic program. ER-specific expression of both SphK1 and SphK2 increases ceramide production associated with apoptosis in cells [77].

In an ex vivo model of colorectal cancer (CRC) using dispensable tissue specimens, S1PR2 was shown to be internalized and to translocate to the ER following S1P engagement, resulting in intracellular uracil build-up that was associated with decreased sensitivity to 5-Fluorouracil (5-FU), one of the standard pillars of chemotherapeutic drugs for the treatment of patients with CRC. Tissue specimens from patients with a poor clinical outcome, as defined by a poor response to 5-FU-based therapy, exhibited increased internalized S1PR2 as compared to S1PR2 expressed on the cell surface [78]. Thus, subcellular S1PR2 may serve as a possible marker for 5-FU chemoresistance.

Other studies examined the expression of the S1P machinery in cancer cell lines. The enzyme sphingosine-1-phosphate lyase (SGPL1) catalyzes the irreversible degradation of S1P. The degradation of S1P decreases cell proliferation and motility [79]. Mutations have also been described in the S1P pathway, e.g., SGPL1 mutations in rhabdomyosarcoma cell lines, resulting in loss of anchorage to the ER and increased cytosolic localization. The mutated SGPL1 failed to decrease S1P levels, thereby enhancing cell migration and colony formation in vitro [80]. This data demonstrated that the S1P pathway operates in a subcellular localization-dependent context and may be explored clinically as a biologically relevant surrogate predictive marker of therapeutic response.

## 4. Association of S1P Components with Diseases

The observation that S1P can feed into the sphingosine pathway led to the concept of a sphingolipid “rheostat”, where the balance between S1P and sphingosine may determine the net effect of S1P concentrations that orchestrate cell fate of transformed and non-transformed cells and, subsequently, cancer progression [45,63].

The production and export of S1P to the TME regulates interactions between cancer cells, immune cells, and mesenchymal cells (Figure 3) [54]. S1P may act as a double-edged sword: S1P may show either anti-cancer- or pro-cancer-directed effects. The activity and net result of S1P result from a complex, regulated network of cellular interactions associated with the timing of S1P production and the balance of intracellular and extracellular S1P concentrations [50]. Clinically and biologically relevant research addressing S1P:S1PR physiology requires very well-defined model systems, either preclinical models or ex vivo tissue explants (e.g., thick tumor sections or organoids).

### 4.1. Evidence of Pro-Tumorigenic Effects of S1P

S1PR1 expression has been associated with a pro-tumorigenic phenotype, promoting tumor cell migration, invasion, proliferation, and neovascularization across several cancer types [55]. SphK1 activity and S1P production are linked to tumor growth, resistance to apoptosis, tumor angiogenesis, and metastasis. SphK1/2 and S1P are often upregulated in cancer tissue, leading to chemo- and radio-resistance and ultimately resulting in a poor prognosis [45,50,52].

Oncogenic microRNAs upregulate SphK1 expression. Increased SphK1 levels have been reported in cancer of the breast, stomach, lung, brain, colon, liver, kidney, and non-Hodgkin lymphoma [50,54,81]. Increased SphK1 expression has been associated with larger tumor size, reduced survival, higher recurrence, and overall poor prognosis in patients with hepatocellular carcinoma, astrocytoma, or breast cancer. In cervical cancer, increased SphK1 has been linked to tumor invasion and lymph node metastasis [45,63]. Resistance to gemcitabine in pancreatic cancer cell lines has been correlated with SphK1 activity [50,63]. SphK2 has been found to be upregulated in acute lymphoblastic leukemia, facilitating c-MYC expression [45,54].

Alterations in S1P transport have also been described in tumor tissue, with overexpression of ABCC1 and SPNS2, leading to increased S1P export. Hepatocellular cancer with a high S1P transport capacity has been found to be aggressive and highly proliferative, correlating with decreased progression-free and overall survival rates [54,81].

Shifts in S1P levels are not only a consequence of altered SphK expression but also of phosphatases and lyases responsible for its degradation. Reduced expression of sphingosine phosphate phosphatases (SPPs) and downregulation of S1P lyases lead to increased extracellular and intracellular S1P levels, a pattern frequently observed across a broad spectrum of cancers, including CRC. Shifts in the levels of phosphatases and lyases that affect S1P have also been associated with increased resistance to chemotherapy [45,50,54,55].

S1P secreted by tumor cells can act within the TME in an autocrine manner, promoting growth, survival, motility, and metastasis. S1P may also act in a paracrine manner, inducing endothelial adhesion molecules and angiogenesis, and regulating the complex network of tumor–stromal and tumor–immune cell interactions [45,50,52].

S1P, produced and released by cancer cells, may act as a “come and get me” signal, attracting monocytes and macrophages. In macrophages, S1P induces an IL-10- and IL-4-producing M2 phenotype. This typically anti-inflammatory phenotype may ultimately suppress the development of an effective anti-cancer-directed immune response (Figure 3) [54].

A recently described mechanism of S1P-mediated immunosuppression involves tumor-derived extracellular vesicles (EVs). In a murine ovarian cancer model, EVs can transport SphK1 into the tumor microenvironment, increasing extracellular S1P. Elevated S1P was linked to T-cell exhaustion and to transcriptional upregulation of PD-L1 on tumor cells via E2F1. Pharmacologic SphK1 inhibition (PF-543) improved T-cell-mediated cytotoxicity, and PF-543, when combined with anti-PD-1, reduced tumor burden and metastasis more effectively than PF-543 alone in vivo [82].

S1PR1 appears to use distinct cellular activation pathways depending on tumor histology, suggesting that caution should be exercised when predicting the action of S1P without considering the tissue-associated context. S1PR1 signaling activates transcription-3 (STAT3) in the TME. However, STAT3 is also a transcription factor for S1PR1: this creates a loop that increases IL-6 expression, a pro-inflammatory cytokine usually associated with tumor progression. STAT3 is also a direct regulator of Th17 effector functions, which, in concert with IL-6, could increase the number and activity of Th17 cells (Figure 3) [39].

The S1PR:S1P axis is tissue- and cancer-specific. For instance, increased S1PR1 expression in glioblastoma cells activates the urokinase plasminogen activator (uPA), which potentiates tumor cell invasion into healthy tissue [83]. In nephroblastoma, S1PR1 signaling leads to tumor cell migration and invasion via the PI3K and Rac pathways [84]. In fibrosarcoma and Hodgkin lymphoma, S1PR1 activates ERK, thereby enhancing tumor cell survival and promoting tumor cell migration [85,86]. Thus, the S1P:S1PR axis is tissue-context-dependent, including the interaction of TILs with cancer and non-transformed cells.

### 4.2. Evidence of Anti-Cancer Effects Mediated by S1P

Angiogenesis is a key player in tumor growth and metastasis development in several solid tumors. Compared with physiological angiogenesis, tumor angiogenesis has been shown to be dysfunctional. To secure growth, cancer cells may develop vasculogenic mimicry (VM), thereby facilitating neovascularization. This vessel-like network is composed of tumor cells lining channels that co-express tumor and endothelial markers. Such networks allow the tumor to be independent of the physiological healthy angiogenic matrix and are often associated with a poor clinical prognosis. In breast cancer, the axis among SphK1, S1P, and S1PR1 regulates this vascular process and serves as a prognostic factor. In studies using breast cancer cell lines, S1PR1 expression inhibited 3D channel formation, leading to lower VM and higher endothelial-dependent vessel formation. Furthermore, S1PR1 overexpression led to reduced tumor cell growth, associated with improved prognosis of patients with breast cancer [87]. It is unclear until now whether these effects are cancer-type-specific, and a broader spectrum of cancer types needs to be examined.

### 4.3. Association of S1P:S1PR1 with the CXCR4:CXCL12 Axis

#### 4.3.1. CXCR4/CXCL12 Axis

Both the S1P:S1PR1 and the CXCR4:CXCL12 axis are wired with the G-protein-coupled receptor (GPCR) pathways and regulate how T-cells move between tissues and the circulatory system. Both pathways lead to downstream signaling of PI3K/Akt/mTOR, MAPK/ERK, and JAK/STAT [88,89].

S1P:S1PR1 acts as an exit signal from the thymus into the circulation, allowing T-cells to patrol for activating cues and to migrate into the tissues. On the other hand, CXCR4:CXCL12, in the absence of disease, traps cells in the bone marrow via CXCR4 expressed on T-cells binding to CXCL12 produced by bone marrow stromal cells, osteoblasts, and endothelial cells. Both axes can be hijacked by cancer cells, creating an immune-excluded environment that prevents effective anti-tumor T-cell responses. High expression of CXCL12 in the tumor stroma leads to the entrapment of CXCR4^+^ T-cells, which cannot infiltrate the peritumoral area, thereby contributing to tumor progression. This axis also drives the recruitment of cancer-associated fibroblasts, creating a fibrotic barrier that impedes tumor infiltration by T-cells [88,90,91]. S1P:S1PR1 can be used to attract macrophages, amplifying an anti-inflammatory response that may contribute to tumor progression [45].

The same hijacking system can occur in the context of cellular metabolism, with CXCR4:CXCL12 allowing cancer cells to adapt to metabolic stress, associated with “quiescent survival” and a “drug-tolerant” state during chemotherapy [92]. Here, the S1P:S1PR1 axis can also interfere with HIF-1α induction, allowing cancer cells to overcome hypoxic conditions [66].

#### 4.3.2. PD-1/PD-L1 Axis

While S1P:S1PR1 orchestrates T-cell trafficking and T-cell fitness, the PD-1:PD-L1 axis functions as an immune checkpoint [93]. High S1PR1 expression is associated with a T-cell stem-like progenitor phenotype, while high PD-1 expression is associated with an “exhausted” and/or activated T-cell phenotype, immune cells that will eventually lose functional capacity in anti-cancer-directed immune responses. PD-1 signaling shifts T-cells away from glycolysis toward fatty acid oxidation, often limiting their energy, while S1P:S1PR1 sustains high energy demands, sustaining cell activity and T-cell fitness [94]. Thus, the S1P:S1PR1 and the PD-1:PD-L1 axes appear to be on opposite sides of the cell activation/exhaustion spectrum.

## 5. Modulation of S1P and Therapeutic Implications

S1P is one of the most analyzed sphingolipid-derived metabolites shaping the TME [38,45,55]. The balance between S1P, ceramide, and sphingosine is critical for the cell fate of tumor cells as well as immune cells that may contain or eradicate tumor cells [95]. Therefore, a study of S1P modulation requires consideration of both interactions: S1P with tumor cells and S1P with immune cells. It has been hypothesized that modulation of the intricate S1P balance (either with S1PR agonists or antagonists) could lead to a more tailored therapy that addresses pro- and anti-apoptotic factors in both tumor cells and immune cells [50,55].

A study by Visentyn and coworkers (2006) validated S1P-specific antibodies that reduced tumor progression in murine models, demonstrating anti-angiogenic and anti-tumorigenic effects [96].

Studies using various cancer cell lines showed that inhibiting SphK1 significantly reduces tumor cell viability and increases sensitivity to chemotherapy and radiotherapy. SphK1 inhibition shifted the ceramide/S1P ratio, allowing the apoptotic effects of ceramide to outweigh the anti-apoptotic activity of S1P [50,63,97].

S1P and S1P receptor modulation have also been identified as viable targets for other immune-related diseases, i.e., multiple sclerosis, rheumatoid arthritis, systemic lupus erythematosus, psoriasis, atopic dermatitis, colitis, and Crohn’s disease, with the clinical development of various therapeutic compounds [98]. Currently, Fingolimod, also known as FTY720 or Gilenya, is approved for the treatment of patients with multiple sclerosis by inhibiting lymphocyte migration and reducing clinical relapse [99]. Since anti-cancer-directed immune responses may be viewed as fine-tuned autoimmune responses directed against antigenic targets preferentially expressed by transformed cells, lessons learned from clinical trials in autoimmune diseases may help elucidate the complex function of the S1P:S1PR axis in patients with cancer.

### 5.1. Candidate S1PR:S1P Delivery Systems to Tumor Cells

S1P and its receptor S1PR were studied in several preclinical tumor models to better understand the complex role of the S1P:S1PR axis in anti-tumor immune effector and memory immune responses. For example, S1PR1 knockout by CRISPR/Cas9 gene editing in a murine ovarian cancer model showed decreased tumor volume compared to the control group [100]. In a squamous cell cancer model, S1PR1 expression was knocked down by plasmid-mediated siRNAs. The group with lower S1PR1 expression showed a slower tumor growth rate as compared to the control group. In contrast, S1PR1 overexpression was associated with faster tumor growth [101]. These studies suggested that the S1PR:S1P axis is a viable target for therapeutic interventions aimed at downregulating S1PR to reduce tumor growth, thereby enabling more effective immune recognition and ultimately tumor containment or eradication by the immune system. Since naked siRNAs are rapidly degraded by nucleases, coupling anti-S1PR1 molecular targeting to nanoparticles may increase the likelihood of safe drug delivery. Currently, there are several options for coupling biological compounds to lipid-based conjugates, including carbohydrate-mediated targeting, antibody conjugation, and peptide conjugation [102,103].

### 5.2. Candidate S1P:S1PR Delivery to Immune Cells

Knocking out S1PR1 in T-cells using an adoptive T-cell transfer model resulted in an accumulation of T-reg cells in lymphoid tissues. Because T-cells are incapable of sensing the physiological S1P gradient (due to the lack of the receptor), decreased immune cell infiltrates were observed. S1PR1 knockout resulted in decreased immune cell infiltrates in the TME [104]. A separate murine study targeting S1PR1 showed that T-cells were more susceptible to pro-apoptotic stress cues than the control group, while humoral immune responses were impaired [105]. Both studies underscore the complex function of S1PR1:S1P and warrant a targeted, “smart” tissue-context-dependent modulation of S1P with exquisite specificity for delivery to the target cells of interest.

## 6. Clinical Trials

As of early 2026, ClinicalTrials.gov lists multiple clinical trials that either modulate the S1P axis directly (e.g., S1PR modulation with fingolimod or sphingosine kinase inhibition with safingol or opaganib). We also list non-interventional, S1P-related biomarker studies. (Table 1). Since S1PR1 functional antagonists can cause lymphopenia, trial interpretation should explicitly report immune cell counts and tumor immune infiltration alongside tumor-centric endpoints.

The clinical development of S1P-targeted agents should not be interpreted as a single therapeutic class, because anti-S1P antibodies, sphingosine kinase inhibitors, and S1PR functional antagonists are expected to affect different biological compartments. Anti-S1P antibodies primarily neutralize extracellular S1P and may affect angiogenesis, tumor cell survival, and immune suppression. SphK inhibitors reduce S1P generation, but their effects may vary depending on whether SphK1 or SphK2 is dominant in a tumor or immune cell context. S1PR modulators, including fingolimod, can affect tumor cell signaling and also redistribute lymphocytes, thereby altering immune cell availability. Therefore, efficacy is likely to differ between angiogenic tumors such as renal cell carcinoma, immune-responsive tumors such as melanoma or some lung cancers, immune-cold or myeloid-rich tumors such as glioblastoma and pancreatic cancer, and gastrointestinal tumors in which S1P signaling may be linked to chemotherapy resistance. The metastatic renal cell carcinoma experience with the anti-S1P antibody sonepcizumab illustrates the challenge of evaluating S1P blockade as monotherapy. The phase 2 trial did not meet its primary 2-month progression-free survival endpoint, although safety was acceptable and overall survival appeared encouraging. This result does not necessarily invalidate S1P as a target but suggests that patient selection and combination strategy may be critical. In that study, patients were not selected for high S1P, SphK1/2 expression, S1PR expression, or angiogenic phenotype, and the trial was not designed to distinguish T-cell-inflamed, immune-excluded, or immune-cold tumors. Similarly, the phase I opaganib/ABC294640 study in advanced solid tumors demonstrated feasibility and pharmacodynamic modulation of plasma S1P levels, but showed only limited single-agent anti-tumor activity across a heterogeneous population. In that trial, plasma S1P decreased during the first 12 h after dosing and returned toward baseline by 24 h, suggesting that plasma S1P can serve as a pharmacodynamic marker but is not necessarily a sufficient predictor of tumor response without paired tumor and immune analyses [106,107,108].

A major limitation of the current clinical trial landscape is the lack of systematic TME stratification. In immunologically “hot” tumors, where CD8^+^ T-cells are already present but functionally restrained, S1P-targeted therapy may be most rational when combined with immune checkpoint blockade or when directed toward tumor cell SphK1/S1P-driven immune escape. In contrast, in immune-cold or immune-excluded tumors, S1P axis modulation may need to be combined with strategies that promote immune priming, T-cell recruitment, myeloid reprogramming, or adoptive cellular therapy. This distinction is important because systemic S1PR1 functional antagonism may simultaneously inhibit tumor-promoting S1P signals and reduce lymphocyte recirculation or tumor entry. Thus, the same drug class could have opposing effects depending on whether the therapeutic goal is to inhibit tumor cell S1P signaling, preserve circulating lymphocytes, retain activated T-cells within tumor tissue, or improve T-cell infiltration into an immune-cold lesion. Future trials should therefore integrate baseline and on-treatment biomarkers rather than relying only on radiographic endpoints. Recommended biomarkers include: plasma and tumor S1P levels; SphK1, Sphk2, SGPL1, SPNS2, MFSD2B, and S1PR1-5 expression in tumor and stromal compartments; CD8/T-reg ratio; CD69, CD103, and S1PR1 expression on CD4^+^ and CD8^+^ TIL; PD-1/PD-L1 and IFN-γ-related T-cell-inflamed signatures; M2-like macrophage markers such as CD163, MRC1/CD206, ARG1, and IL-10; and spatial localization of CD8^+^ T-cells relative to tumor nests, vasculature, stromal barriers, and tertiary lymphoid structures. These readouts would help distinguish pharmacodynamic target engagement from clinically meaningful TME remodeling [62,109,110].

As summarized in Table 1, current oncology-related S1P studies include interventional trials testing S1P axis modulators and observational studies using S1P-related biomarkers. These studies differ substantially in therapeutic intent. Some trials assess the direct anti-tumor effects of S1P axis modulation, such as fingolimod in lung cancer and ABC294640/opaganib in advanced solid tumors. Others use S1P as a pharmacodynamic or prognostic biomarker, for example, in studies of glioblastoma or treatment-related toxicity. Therefore, these trials should be separated conceptually into: (i) therapeutic S1P axis targeting (NCT06424067, NCT03941743, NCT02490930, NCT01488513, and NCT02229981), (ii) supportive care or lymphocyte-trafficking interventions (NCT05291286), and (iii) biomarker-only studies (NCT04283539, NCT04657146, and NCT06348693). This distinction is important because a negative or inconclusive result in one category may not predict failure in another category.

The study NCT02229981, using a sphingosine kinase inhibitor to assess safety and dosing, was withdrawn due to insufficient patient recruitment.

NCT05291286, NCT03941743, and NCT02490930, even though they have been completed, have not yet shared their results publicly.

NCT01488513 published results in 2017. The most common drug-related toxicities were nausea, vomiting, and fatigue, which were found to be tolerable, using the recommended dose for the phase II clinical trial. Higher doses led to intolerable non-cardiac chest discomfort and choking sensations. The use of the Sphk2 inhibitor appeared to reduce plasma S1P levels in patients 12 h after administration, with recovery to baseline S1P levels reported 24 h post-administration. No consistent long-term changes in S1P levels were reported. Regarding anti-tumor efficacy, one patient with cholangiocarcinoma showed a partial response (for a total of 18 cycles) before developing progressive disease; one patient with metastatic urothelial carcinoma ultimately developed progressive disease after 12 cycles; one patient with cholangiocarcinoma was removed from the study in cycle 3 due to toxicity; and four patients came off the study for progressive disease after 8 weeks. The remaining eight patients showed progressive disease. Since there is a redundancy in the phosphorylation of sphingosine into S1P (either by SphK1 or SphK2), one may argue that S1P levels can be restored to physiological levels by the compensation of SphK2 inhibition by a more active SphK1 (although this would require confirmation). Thus, the best approach may lie in targeting a component of the S1P:S1PR1 axis that is not redundant, for example, S1PR1. The systemic drug-related toxicities might be related to the systemic delivery of the drug, which could be overcome by an, although challenging, targeted delivery. In the case of local delivery, the entire cellular composition of the TME may be affected by SphK2 inhibition and not only cells where inhibition S1PR1:S1P is thought to be beneficial: a rather common challenge associated with local (non-targeted) delivery of drugs that may exhibit beneficial or deleterious outcomes due to the complex cellular composition of pro- and anti-tumor-directed effects in the TME.

Published clinical experience with S1P-targeted therapeutics in oncology remains limited. For example, a phase 2 study of the anti-S1P monoclonal antibody sonepcizumab in patients with metastatic renal cell carcinoma did not meet its primary endpoint of progression-free survival but showed an acceptable safety profile; this may motivate future combinatorial therapeutic strategies. Early-stage phase I trials of the SK2 inhibitor opaganib (ABC294640) demonstrated feasibility and pharmacodynamic target engagement in patients with advanced solid cancer [106,108].

Several mechanisms may explain why S1P-targeted therapies have shown limited single-agent efficacy in oncology. First, S1P signaling is redundant: S1P can be generated by both SphK1 and Sphk2, exported by multiple transporters, carried by distinct extracellular chaperones, and sensed by five receptors with partially overlapping but nonidentical functions. Second, plasma S1P modulation may not reflect intratumoral S1P biology, particularly when tumor cells, stromal cells, endothelial cells, platelets, and macrophages contribute to local S1P gradients. Third, S1P blockade may affect immune and tumor compartments in opposite directions. For example, reducing tumor cell S1P signaling may decrease immune escape, while systemic S1PR1 functional antagonism may impair lymphocyte recirculation or tumor infiltration. Fourth, resistance may be driven by compensatory immune-suppressive circuits, including PD-1/PD-L1 signaling, T-reg enrichment, Th17-driven inflammation, and M2-like macrophage polarization. These considerations support rational combination strategies. In immune-hot tumors with pre-existing TILs, S1P axis targeting may be most useful as a partner for immune checkpoint blockade, particularly when biomarkers indicate SphK1-high, PD-L1-high, T-reg-rich, or M2-macrophage-rich disease. Preclinical melanoma data show that tumor SphK1 can mediate resistance to anti-PD-1 and anti-CTLA-4 therapy, and that SphK1 targeting can increase the intratumoral CD8/T-reg ratio and improve checkpoint inhibitor efficacy. In ovarian cancer models, tumor-derived extracellular vesicles carrying SphK1 increased S1P in the TME, promoted T-cell exhaustion and PD-L1 expression, and the combination of SphK1 inhibition with anti-PD-1 reduced tumor burden and metastasis more effectively than SphK1 inhibition alone. In tumors with poor endogenous T-cell infiltration, S1P modulation may need to be combined with adoptive cell therapy, tumor vaccination, radiation, or other priming strategies to introduce or expand tumor-reactive T-cells. In this setting, ex vivo modulation of the S1P pathway during cell manufacturing may be preferable to systemic S1PR blockade, because it can improve T-cell fitness while avoiding unwanted lymphocyte sequestration [62,82,109,111].

## 7. Conclusions

S1P and its S1PR1-5 receptors are biologically and clinically relevant players in orchestrating the TME. S1PR expression, temporospatial concentrations of S1P, and intracellular signaling in cancer cells and immune cells shape anti-cancer-directed immune responses.

Retrospective sample analysis of TIL therapy suggest that a progenitor or stem-like TIL phenotype is associated with favorable clinical outcome. increases tge. These progenitor-like phenotypes are usually defined as CD8^+^ and CD69^−^. Logically, due to the existence of a mutual exclusivity in the expression of CD69 and S1PR1, these cells are S1PR1^+^. Since CD69 is characteristic of tissue-entrapped cells (or tissue-homing cells), the absence of CD69 and the expression of S1PR1 allow T-cells to egress from lymphoid organs and recirculate, a profile necessary for a long-term protective immune response.

The premise that S1P and S1PR1 can be clinically relevant is also supported by the observation that CD8^+^ TILs are susceptible to the S1P:S1PR1 axis, particularly with respect to T-cell migration. Furthermore, the expression of S1PR1 in “enhanced” TIL sustains the high energetic demand characteristic of precursor-like T-cells, ensuring T-cell activity, longevity, and fitness: S1P increases mitochondrial fitness, a biologically relevant status of TIL residing in an adverse TME.

The hypothesis that S1PR1^+^ TILs mediate clinically relevant immune responses warrant further research and may aid in enriching for anti-cancer-directed precursor T-cells recognizing cancer cells, studies that are ongoing in our laboratory.

Future directions: Dissecting the S1P axis in cancer likely requires spatially resolved, multi-omics profiling to capture tumor microenvironment heterogeneity, including tissue-resident versus circulating T-cell states and tertiary lymphoid structure programs. Computational frameworks that integrate bulk and single-cell datasets can support hypothesis generation and biomarker discovery. In parallel, tumor-targeted delivery systems for immunomodulatory agents (e.g., nanoparticle- or exosome-based) could localize S1P pathway modulation while minimizing systemic lymphopenia [112,113].

## Figures and Tables

**Table 1:** Summary of clinicals trials involving S1P listed in ClinicalTrials.gov in January 2026. (NA—non applicable).

Trial Identifier	Disease	Phase/Status	Drug	Outcome Measure
NCT06348693	Glioblastoma	Observational recruiting	NA	Evaluation of prognostic and predictive role of S1P levels on tumor chemoresistance. Time frame of 3 years.
NCT04283539	Immune-related cutaneous adverse events in patients with solid tumors treated with checkpoint inhibitors	Observational unknown status	Systemic corticosteroid or biologicals.	S1P levels in the skin and circulating S1P as secondary outcome measures. Time frame of 30 days and 12 months.
NCT05291286	Chemotherapy-induced peripheral neuropathy	Phase 1 completed	BXQ-350 or placebo administered for six months.	S1P levels as primary outcome measures. Time frame of 6 weeks.
NCT04657146	Glioblastoma	Observational suspended	NA	S1P levels as a primary outcome measure and their correlation with blood and bone marrow T-cell counts over the course of treatment. Time frame of 2 years.
NCT01488513	Pancreatic cancer	Phase 1 completed	Sphingosine kinase-2 inhibitor—ABC294640 (oral). Administered twice daily on days 1–28. Courses repeat every 28 days.	S1P levels as primary outcome measures. Anti-tumor activity of the compound, safety, maximum tolerated dose, and the dose-limiting toxicities as primary outcome measures.
NCT03941743	Breast carcinoma	Phase 1 completed	Fingolimod (oral) administered one day before chemotherapy, on the day of chemotherapy, and 1 day after chemotherapy for 12 weeks	Prevention of chemotherapy-induced peripheral neuropathy as a primary outcome measure.
NCT02490930	Glioblastoma anaplastic astrocytoma	Phase 1 completed	Fingolimod (oral) administered 1 week prior to the initiation of concurrent radiation.	Incidence of infections attributable to fingolimod-induced lymphopenia as a primary outcome measure
NCT02229981	Diffuse large B-Cell lymphoma Kaposi Sarcoma	Phase 1 and 2 withdrawn (lack of recruitment)	Sphingosine kinase-2 inhibitor—ABC294640 (oral).	Safety, maximum tolerated dose, and dose-limiting toxicities of the compound as primary outcome measures. S1P levels in plasma as secondary outcome measures.
NCT06424067	Non-Small Cell and Small Cell Lung Cancer	Phase 2 recruiting	Fingolimod (oral).	Safety, maximum tolerated dose, progression free survival, and overall survival. Time frame of 6 months.

## Data Availability

No new data were created or analyzed in this study.

## Abbreviations

The following abbreviations are used in this manuscript:

ACT	adoptive cell therapy
ABC	ABC transporter family
CD	cluster of differentiation
Akt	protein kinase B
MHC	major histocompatibility complex
HLAs	human leucocyte antigens
IFN	interferon
IL	interleukin
FDA	Food and Drug Administration
TILs	tumor-infiltrating lymphocytes
NA	non-applicable
KRAS	Kirsten rat sarcoma virus
DNA	deoxyribonucleic acid
NK	natural killer
BCL-2	B-cell lymphoma-2
BAD	B-cell lymphoma-2-associated death promoter
BAX	B-cell lymphoma-2-associated X protein
STAT	signal transducer and activator of transcription
GPCRs	G-protein-coupled receptors
MART-1	melanoma antigen recognized by T-cells 1
NY-ESO-1	New York esophageal squamous cell carcinoma 1
PRAME	preferentially expressed antigen of melanoma
S1P	sphingosine-1-phosphate
S1PR	sphingosine-1-phosphate receptor
TME	tumor microenvironment
TCR	T-cell receptor
EDG-1	endothelial differentiation gene-1
Ras	rat sarcoma
ERK	extracellular signal-regulated kinases
NLRP3	NLR family pyrin domain containing 3
PI3K	phosphoinositide 3-kinases
mTOR	mammalian target of rapamycin
PLC	phospholipase C
MAPK	Mitogen-activated protein kinases
cAMP	cyclic adenosine monophosphate
PINK	PTEN-induced kinase 1
T-regs	regulatory T-cells
ERM	ezrin–radixin–moesin
Rho	Ras homologous
JNK	Jun N-terminal kinase
SAPK	stress-activated protein kinase
TRAF	tumor necrosis factor-associated factor
PKC	protein kinase C
TNF	tumor necrosis factor
SphK	sphingosine kinase
HIF	hypoxia-inducible factor
HDAC	histone deacetylases
Drp1	dynamin-related protein 1
PHB2	prohibitin
ATP	adenosine triphosphate
BOK	BCL-2-related ovarian killer
ER	endoplasmic reticulum
ROS	reactive oxygen species
CRC	colorectal cancer
5-FU	5-Fluorouracil
SGPL1	sphingosine-1-phosphate lyase
mRNA	messenger RNA
RNA	ribonucleic acid
SPNS2	SPNS lysolipid transporter 2
SPPs	sphingosine-1-phosphate phosphatase
uPA	urokinase plasminogen activator
VM	vascular mimicry
